# An Intelligent Fault Diagnosis Method for Bearings with Variable Rotating Speed Based on Pythagorean Spatial Pyramid Pooling CNN

**DOI:** 10.3390/s18113857

**Published:** 2018-11-09

**Authors:** Sheng Guo, Tao Yang, Wei Gao, Chen Zhang, Yanping Zhang

**Affiliations:** School of Energy and Power Engineering, Huazhong University of Science & Technology, Wuhan 430074, China; levykwok@hust.edu.cn (S.G.); gw@hust.edu.cn (W.G.); zhangchen710@yeah.net (C.Z.); zyp2817@hust.edu.cn (Y.Z.)

**Keywords:** convolutional neural network, spatial pyramid pooling, fault diagnosis, bearing, wavelet transform

## Abstract

Deep learning methods have been introduced for fault diagnosis of rotating machinery. Most methods have good performance when processing bearing data at a certain rotating speed. However, most rotating machinery in industrial practice has variable working speed. When processing the bearing data with variable rotating speed, the existing methods have low accuracies, or need complex parameter adjustments. To solve this problem, a fault diagnosis method based on continuous wavelet transform scalogram (CWTS) and Pythagorean spatial pyramid pooling convolutional neural network (PSPP-CNN) is proposed in this paper. In this method, continuous wavelet transform is used to decompose vibration signals into CWTSs with different scale ranges according to the rotating speed. By adding a PSPP layer, CNN can process CWTSs in different sizes. Then the fault diagnosis of variable rotating speed bearing can be carried out by a single CNN model without complex parameter adjustment. Compared with a spatial pyramid pooling (SPP) layer that has been used in CNN, a PSPP layer locates as front layer of CNN. Thus, the features obtained by PSPP layer can be delivered to convolutional layers for further feature extraction. According to experiment results, this method has higher diagnosis accuracy for variable rotating speed bearing than other methods. In addition, the PSPP-CNN model trained by data at some rotating speeds can be used to diagnose bearing fault at full working speed.

## 1. Introduction

As most rotating machinery are the key equipment in production and work in a high-speed rotating environment, their failures will cause major economic losses and safety accidents. Therefore, it is important to detect equipment failure as soon as possible. An intelligent fault diagnosis method has the motivation that it can detect the fault of operating machinery in real time without manual operation. With the popularization of online vibration monitoring systems, manufacturers have accumulated a large amount of data that can support intelligent fault diagnosis methods. Many intelligent fault diagnosis methods have been proposed to diagnose bearing faults [1,2,3,4,5]. However, most of them use a simple classifier and focus on fault feature extraction algorithms. When these methods are used to diagnose bearings with complex operating conditions, the simple classifier cannot process large amounts of monitoring data and can easily cause over fitting.

As the advanced representation of intelligent algorithms, deep learning methods have greatly changed our daily life. They are successfully applied in many different areas such as computer vision, object detection, natural language processing, and even disease diagnosis [6,7,8,9,10]. Deep learning methods can recognize high-dimensional complex input, get rid of the reliance on signal processing techniques and hand-engineered feature extraction algorithms. Unlike most previous artificial intelligent methods that can only process one-dimensional hand-engineered features [11], they can process the two-dimensional results of some basic signal processing methods directly. With these advantages, deep learning methods have been introduced to the fault diagnosis of rotating machinery, such as convolutional neural network (CNN), deep belief nets (DBN), and recurrent neural networks (RNN) [12,13,14,15,16,17]. Most methods perform well when dealing with bearing data at a certain rotating speed. However, in practical use, most machinery, such as wind turbines, pumps, and fans, have varying rotating speed. The performance of the methods has not been verified when processing data with varying rotating speed. Liu et al. [18] propose a dislocated time series CNN method for bearing diagnosis and apply it to varying rotating speed data by testing different network parameters to achieve the best result. However, the number of parameters that can be tested is limited, and the method is difficult to realize in practical applications. Meanwhile, in practical use, faults may not happen fully in all working speeds. Then there will not be enough fault data that covers full working speeds. Therefore, there needs to be a new deep learning fault diagnosis framework which can deal with data with varying rotating speed without complex parameters attempt. In addition, it needs to be able to realize diagnosis in all working speed based on limited fault data.

The continuous wavelet transform (CWT) has been proved to be a useful method to analyze vibration signals [19,20,21]. As a time-frequency analysis method, the result contains the complete time-frequency domain information of the vibration signal and avoids information loss of the original signal. In addition, it is suitable for detecting bearing faults which is usually presented as shock signals. When used in fault diagnosis of bearings, it has advantages compared with some other time-frequency methods, such as Short-time Fourier Transform (STFT), Discrete Wavelet Transform (DWT), Wavelet Package Decomposition (WPD) and Empirical Mode Decomposition (EMD). 

Short-time Fourier Transform is the time-frequency transform based on Fourier transform. Because the window size is fixed, it only applies to stationary signals with small frequency fluctuations. In addition, the result is susceptible to noise interference. DWT is the discretization of the scale and displacement of CWT according to the power of 2. It retains less time-frequency information than CWT and may lose the key information near fault characteristic frequencies of bearing. WPD is an improved method of CWT. It provides a more detailed decomposition of high-frequency components. The fault characteristic frequencies of a bearing are usually less than 12 multiples of the rotating frequency. With the high sampling frequency, all the information near fault characteristic frequencies can be got by CWT. There is no need to use WPD, which is a more time-consuming method than CWT. Empirical Mode Decomposition decomposes the signal based on the time scale characteristics of the signal itself. Without a preset basis function, the location of the fault feature is uncertain in EMD when it applies to signals of different sensors. Therefore, when used for deep learning method, CWT, which uses a fixed wavelet basis function to decompose all the signals, is a better choice.

However, in most cases, a feature exaction method is used to exact a one-dimensional vector from the two-dimensional continuous wavelet transform coefficients. It may result in the loss of key fault information. Continuous wavelet transform scalogram (CWTS) contains all the continuous wavelet transform coefficients. It is a two-dimensional matrix which contains the complete time-frequency domain information of the vibration signal. With its powerful image recognition ability, CNN is the most suitable deep learning method to deal with CWTS. When applied to data with varying rotating speed, the CWTSs will have different size if they have the same frequency multiplication range of the rotating frequency. Without a cropping or warping operation, ordinary CNN can only process the input in the same size. Therefore, a CNN with new structure is needed to process CWTSs in different sizes.

To overcome the problems and challenges above, this paper proposes a fault diagnosis method based on continuous wavelet transform and Pythagorean spatial pyramid pooling (PSPP) CNN. This method uses a continuous wavelet transform to decompose vibration signals into CWTS in different scales according to the rotating speed. Using the PSPP strategy, CNN could then process the different size scalograms. Therefore, the fault diagnosis of data at variable rotating speed can be carried out by a single CNN model. The PSPP strategy is an improvement on spatial pyramid pooling (SPP). A PSPP layer can locate as front layer of CNN. Thus, the features obtained by the PSPP layer can be delivered to the convolutional layers for further feature extraction. Experiments are carried out on data from two different testbeds, constant rotating speed data and variable rotating speed data, respectively. The results demonstrate the effectiveness of the proposed approach. The contributions of the proposed approach are as follows:

(1) Compared with features extraction method used before when dealing with continuous wavelet transform coefficients, using a two-dimensional CWTS for fault diagnosis directly can retain the complete time-frequency domain information of signal and avoid the loss of fault information.
(2) A PSPP layer is proposed based on the SPP layer. In contrast with SPP-CNN, PSPP-CNN can place convolutional layers after the PSPP layer for further feature extraction. A PSPP layer can also retain position information of input feature maps. Experiment results show that PSPP-CNN performs better than SPP-CNN.
(3) A CWTS cropping method is presented to crop CWTSs to different sizes according to rotating speed and sample frequency. The objects recognition using CNN is concerned with the shape of the object. However, in signal processing area, the location of the signal features should also be paid attention to. The cropped CWTSs have the same frequency and time domain range. It helps the PSPP-CNN to achieve a more accurate and faster convergence.
(4) The proposed method can process data in different rotating speeds using a single CNN without complex parameter selection. PSPP-CNN trained by data at some rotating speeds can be used to diagnose bearing fault in full working speed. The experiments provide a good result.

The paper is organized as follows. Section 2 presents the fault diagnosis method that combines CWTS and PSPP-CNN for fault diagnosis, with a detailed procedure of the proposed method and SPP, the proposed PSPP layer, and the structure of PSPP-CNN used in this paper. Experimental verification of the method which includes constant rotating speed data and variable rotating speed data is described in Section 3. Finally, the concluding remarks are given in Section 4.

## 2. Proposed Method

**Figure 1:**
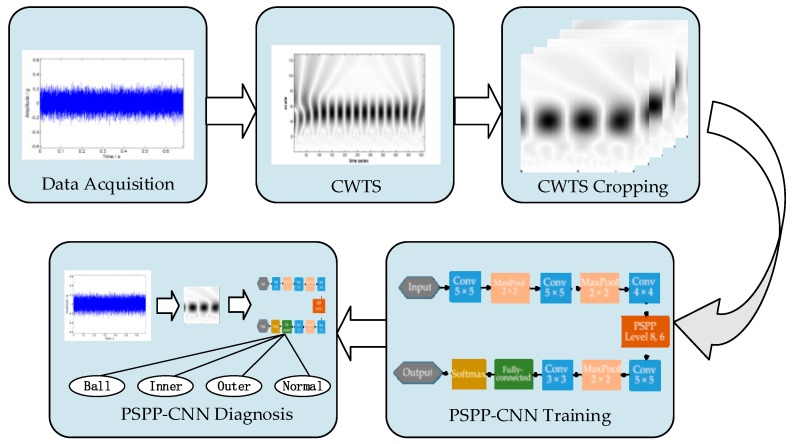
Flow chart of proposed fault diagnosis method. CWTS represents Continuous Wavelet Transform Scalogram, and PSPP-CNN represents Pythagorean Spatial Pyramid Pooling Convolutional Neural Network.

As described above, CNN has been successfully applied to fault diagnosis. However, the proposed fault diagnosis models lack the diagnosis of variable rotating speed data, which has practical engineering value for online diagnosis of variable speed equipment such as the wind turbine. Therefore, this paper proposes a fault diagnosis framework based on CWTS and PSPP-CNN. Figure 1 illustrates the procedure of the proposed method. First, accelerators are used to collect the vibration signals of bearing. Second, continuous wavelet transform is used to decompose vibration signals into CWTSs. Next, as fault characteristic frequencies of bearings are related to rotating speed, the CWTSs are cropped into different sizes according to rotating speed. Then using PSPP strategy, CNN can deal with the input of different sizes. Therefore, the CWTSs in different sizes can be trained using a single PSPP-CNN. Finally, the test signals of bearing with variable rotating speed need to be decomposed into CWTSs with different scale ranges according to rotating speed. Using the CWTSs as the input of the trained PSPP-CNN, fault diagnosis of the signals can be achieved. Details of the main steps of the proposed method are described as follows:

### 2.1. Continuous Wavelet Transform Scalogram

The continuous wavelet transform decomposes a signal in the time-frequency domain by using a family of wavelet functions to obtain feature values. Next, by analyzing the continuous wavelet coefficients or using the classification algorithm, we gain insight about the fault condition of the equipment. The process of continuous wavelet transformation can be described as:

(1) $$\;\Psi_{a,b}\left(t\right)={\left|a\right|}^{-\frac{1}{2}}\Psi \left(\frac{t-b}{a}\right)\;\;a,b\in R\;a\neq 0\;$$

(2) $$\;C_{a}\left(k\right)=\int x\left(t\right){\bar{\Psi }}_{a,b}\left(t\right)dt\;$$

where Ψ*_a,b_*(*t*) is a wavelet function whose shape and displacement are determined by *a*, the scale parameter, and *b*, the translation parameter. *x* is a signal with *m* data points. The wavelet coefficient of *x*(*t*) at the *a*-th scale is *C_a_* (*a* = 1,2,3,⋯,*l*). *k* is time order (*k* = 1,2,3,⋯,*m*). ${\bar{\Psi }}_{a,b}(t)$ is the complex conjugate of the wavelet function at scale *a* and translation *b*.

To show the change of wavelet coefficients intuitively, a CWTS is proposed [22]. The CWTS expresses continuous wavelet coefficients by a two-dimensional image in the time-frequency domain. Put all wavelet coefficients in a matrix ***P*** = [*C*_1_,*C*_2_,⋯,*C_l_*]. The graph of wavelet coefficients matrix ***P*** is called a CWTS. 

**Figure 2:**
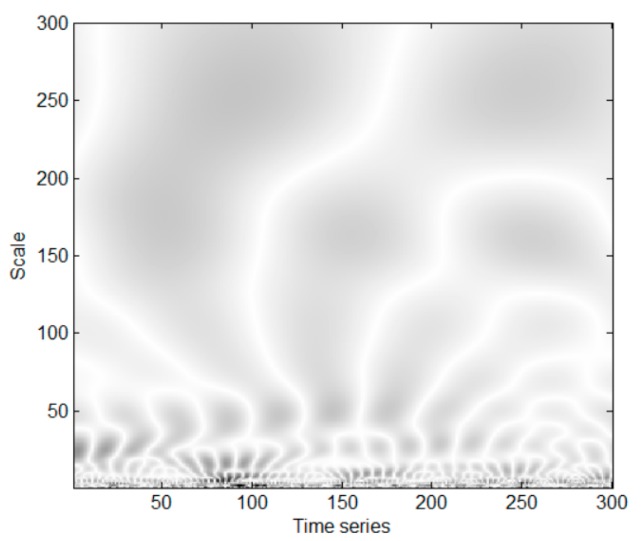
Continuous Wavelet Transform Scalogram (CWTS) of a ball fault bearing signal. The darker pixels correspond to larger wavelet coefficients.

Figure 2 shows the CWTS of a ball fault bearing signal with a 2400 rpm rotating speed sampled at 12 kHz. It is decomposed by the Morlet wavelet from 1 to 300-scale and has 300 data points in time series. The horizontal axis represents the position along the time direction, and the vertical axis represents the scale. Morlet wavelet is chosen as the wavelet used in this paper. Because it has the similar shape with the shock signal caused by bearing faults [23]. In addition, the signal extracted by the Morlet wavelet has the higher energy-to-Shannon entropy ratio than the other common wavelet types. Energy-to-Shannon entropy ratio is an important indicator to measure the fitness of wavelet functions [24].

### 2.2. Continuous Wavelet Transform Scalogram Cropping

The object recognition using CNN is concerned with the shape of the object. If the shape appears in the image, the existence of the object is detected. However, in signal processing area, the axes of images constructed usually have clearly defined meanings. The appearance of the same shape in different location may indicate different fault modes. Therefore, the location of the features should also be paid attention to.

As the vertical axis of CWTS represents the scale, different positions on the vertical axis relate to different frequencies. As we know, the fault characteristic frequencies of bearings are related to rotating speed. If the input CWTSs of PSPP-CNN can be ensued to have the same time domain range and frequency multiplication of the rotating frequency, the fault characteristic of the same fault could appear at the similar position in CWTSs. Thus, the classification of CWTSs will achieve a comparatively accurate result. Therefore, a CWTS cropping step is proposed in this paper.

Suppose a vibration signal *x*(*i*) (*i* = 1,2,…*m*) is collected at a sampling frequency *f* (Hz) with *m* sampling data points. The rotating speed is *n* (rpm), corresponding to a machine rotating frequency $f_{m}\;=\;n/60$. The integer multiple *f* to $f_{m}$ is

(3) $$q\;=⎣\frac{f}{f_{m}}+\frac{1}{2}⎦=⎣\frac{60f}{n}+\frac{1}{2}⎦$$

From Equation (1), we can calculate that the central frequency of a wavelet function is inversely proportional to scale *a*. Suppose $f_{j}\;=\;k/j$, where *f_j_* is the central frequency at scale *j*, and *k* is the proportionality coefficient. According to the Morlet wavelet function,

(4) $$\;k\;=f_{0}\times \;f\;$$

where *f*_0_ is the center frequency of the wavelet function, and the range is from 0.796 to 0.955. In this paper, 0.955 is chosen as *f*_0_. Therefore, we choose scale 1 as the starting scale of cropping which corresponds to a high frequency. To make the end scale the same multiple of the rotating frequency, we choose *q* as the end scale. Scale *q* corresponds to the frequency:

(5) $$\;f_{q}\;=\;k/q=k/{f\;\times \;f}_{m}={\;f}_{0}{\;\times \;f}_{m}\;$$

Thus, *f_q_* is the *f*_0_ multiplication of the rotating frequency. As the fault characteristic frequencies of bearings are larger than the rotating frequency, the cropping from scale 1 to *q* at the scale axis is sufficient for bearing fault diagnosis. The cropped CWTS will contain all the fault characteristic frequencies of bearings needed for analysis.

For the time domain axis, the time of *q* length data points is *t* = *q*/*f* ≈ 60/*n*, which is about the time duration of a rotor rotation cycle. 

Therefore, in this paper, the original CWTS is cropped from scale 1 to *q*, and *q* length in the time domain axis. Thus, the cropped CWTSs with different rotating speeds have the same time domain range and frequency multiplication relative to the rotating speed.

### 2.3. Pythagorean Spatial Pyramid Pooling Convolutional Neural Network Training

#### 2.3.1. Pythagorean Spatial Pyramid Pooling Convolutional Neural Network

A CNN comprises convolutional layers, pooling layers, and fully connected layers. Most CNNs require a fixed input size. So before being sent into the first CNN layer, images need a cropping or warping operation. Convolutional layers and pooling layers do not need a fixed input size. However, the fully connected layers require a fixed input and output size to maintain constant number of the full connections. SPP can pool the mixed-size images into fixed-length outputs, thus meeting the need for fixed inputs in the fully connected layers.

Spatial pyramid pooling (or spatial pyramid matching) was first used in computer vision. Used together with feature extraction and classification algorithms, it has shown good results in image classification [25,26,27], object recognition [28,29,30], semantic concept detection [31], and image memorability [32]. Next, the SPP layer was introduced to CNN to remove the fixed-size input constraint of CNN [33]. The SPP-CNN method has been used in remote sensing hyperspectral image classification [34], handwritten word image categorization [35], and action recognition [36]. According to these applications, SPP is useful in CNN. It can reduce the cropping and warping operations used to fit a fixed-size CNN input, and avoid information loss in the operations.

**Figure 3:**
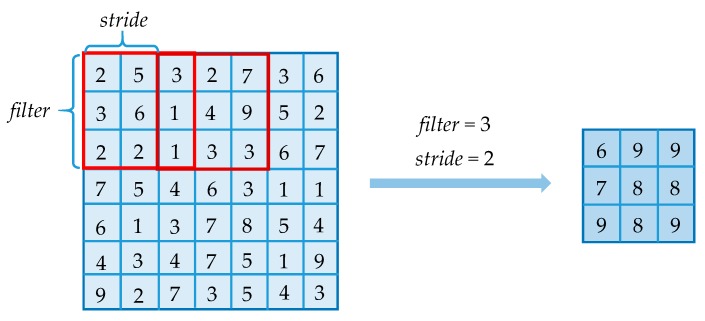
Max pooling process. Get the maximal value in the range of each pooling operation.

The ordinary pooling layer in CNN is used to compress the input feature maps. It helps to reduce the feature maps and simplify the computational complexity of the network. It also extracts the main features from the original maps. There are two general types of pooling operations: average pooling and max pooling. Figure 3 shows an example of max pooling process in CNN, where *filter* is the filter size that indicates the range of pooling operation. *stride* is the space between pooling operations. It is clear that if the input image size changes more than the stride, the output size will change. This will make the classification algorithm impossible to continue.

**Figure 4:**
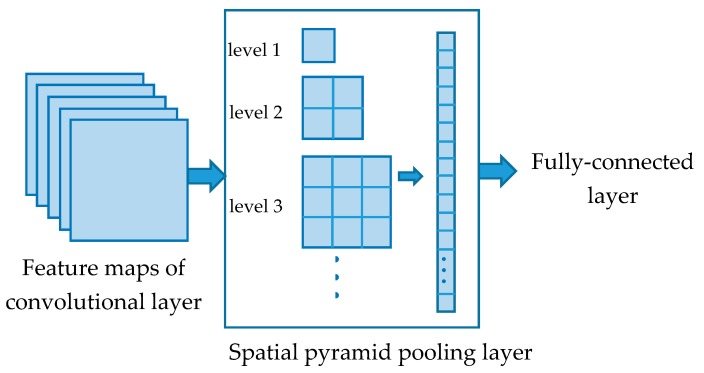
Convolutional neural network (CNN) with a spatial pyramid layer. Each input image is pooled to several levels. The results are transformed into one-dimension vectors to form the spatial pyramid pooling (SPP) output.

To resolve the requirement for a fixed input size, SPP is introduced to CNN as the last pooling layer. As shown in Figure 4, the feature images are pooled to different levels in the SPP layer. We will get an *l × l* size image in level *l*. Thereafter, a fixed-length output can be obtained by *n* level pooling. Feature values $\overset{n}{\underset{i=1}{\sum }}i^{2}$ will be sent into the fully connected layer for classification.

To get an *l* × *l* size image in level *l*, the filter size and stride should change by level. The *filter* and *stride* can be computed by

(6)  filter=[m/l]

(7)  stride=[m/l]

where *m* × *m* is the size of the feature maps from the last layer. Table 1 shows an example of 4-level SPP. Two input images with different size 15 × 15 and 20 × 20 get the same 30 length output by a 4-level SPP layer. If the input size changes, the pooling parameters will change to ensure the outputs have the same length.

Although SPP-CNN has shown good performance in image classification, there are still some problems. As the SPP layer lies at the last, before the fully connected layer in CNN, the outputs of the SPP layer are sent directly into the fully connected layer for classification. However, with no convolutional layer, the features obtained by the SPP layer may not be fully used. In addition, some of the location information will be lost. Meanwhile, the fully connected layer will have a large input matrix. It will greatly increase the connections in fully connected layer. Then there will be much more parameters to be trained. Therefore, a PSPP layer is proposed to make full use of the features and reduce the parameters.

**Figure 5:**
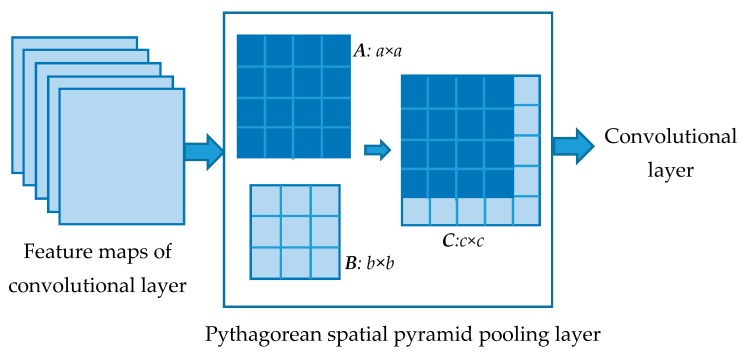
Structure of Pythagorean spatial pyramid pooling (PSPP) layer. It constructs the output using the results of two pooling levels. The position information of the higher-level pooling result ***A*** are retained.

The structure of the PSPP layer is shown in Figure 5. SPP is used to pool the input images into two different levels. Next, the output of the two levels will be used to compose new feature maps rather than a feature vector in the SPP layer. Thus, the output feature maps can be delivered to the convolutional layer for another round of feature extraction. 

To facilitate the composition of two SPP outputs, the pooling levels *a, b* (*a* > *b*) are chosen from the smaller two numbers of the Pythagorean triple. Hence, the output feature maps will have the size of the largest number *c* in the Pythagorean triple. To retain some position information of the feature maps, the composition is processed in the following way. The output matrix of the higher pooling level ***A*** will be retained. Next, the smaller output matrix ***B*** is reshaped as (*c* + *a*) × (*c* − *a*). The reshaped matrix is used to expand ***A*** to ***C*** on the right side and down side.

**Figure 6:**
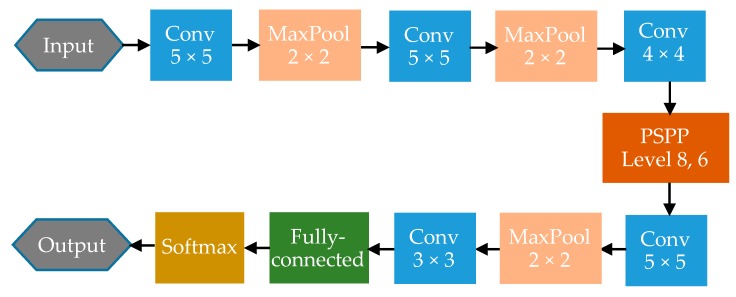
Structure of PSPP-CNN used in this paper. It is the PSPP-CNN used in all the following experiments, as PSPP-CNN has ability to process multi-size input.

Using the PSPP layer in CNN, the fixed input problem can be solved. In addition, the output of the layer are square matrices which can be extracted in the following steps. The structure of the PSPP-CNN used in multi-size training of this paper is shown in Figure 6. Two convolutional layers are added after the PSPP layer for further feature extraction. The convolutional layers will also reduce the size of feature maps. Then the connections in fully connected layer is reduced.

It is recommended that the PSPP layer be in the middle layers of PSPP-CNN. As the PSPP layer has a larger feature reduction than normal 2 × 2 or 3 × 3 max pooling, the ahead position PSPP layer will lead to the premature loss of features. The PSPP layer at the back position is more like an SPP layer without enough convolutional layers to use the features obtained.

The size of convolutional and pooling kernels is changeable according to the input image size. However, big kernel size may result in information loss and increase computational complexity. Therefore, we choose to add more convolutional layers when processing large input images. 

#### 2.3.2. Pythagorean Spatial Pyramid Pooling Convolutional Neural Network Training Method

According to the previous description, the forward process is easy to realize. The filter size and stride can be pre-computed before the pooling. However, the back-propagation process in PSPP-CNN training requires some strategy.

When a back-propagation result is received from the last layer, the result is first divided to levels in the same order as the forward process. Next, the result in each PSPP level is restructured as a square matrix. The square matrices apply back-propagation separately. Thus, 2 back-propagation matrices are obtained. Hence, there are two ways to calculate the back-propagation matrix of an PSPP layer: (1) the mean of 2 back-propagation matrices, and (2) the weighted mean of 2 back-propagation matrices according to the level. The calculations can be presented by (8) and (9)

(8) $$\;d(i)=\frac{1}{2}\overset{2}{\underset{k=1}{\sum }}d_{k}(i+1)\;$$

(9) $$\;d(i)=\frac{1}{\sum_{k=1}^{2}k^{2}}\overset{2}{\underset{k=1}{\sum }}k^{2}d_{k}(i+1)\;$$

where *d*(*i*) is the back-propagation matrix of layer *i* in CNN, *d_k_*(*i* + 1) is the level *k* back-propagation matrix of layer *i* + 1 which is an PSPP layer. The two methods are tested using the same CNN structure, samples, and learning rate. The samples are part of the data used in Section 3.1. The results are listed in Table 2. Training error rate less than 0.05% is consider as achieving convergence.

As shown in Table 2, the two methods have similar accuracy and during time of each training step. However, the back-propagation using the second method has a faster convergence rate. This is because that the high-level pooling in PSPP reserves more features from feature maps. Therefore, the high weight of high-level pooling will lead the training to the right direction.

When PSPP-CNN is applied to multi-size images training, an important problem is the training order of the multi-size samples. To prevent the network from fitting a single image size, the multi-size samples in our work will be trained by turns. After all the samples of one size are trained, we will switch to another size. When the training error rates of samples in each size are less than 0.1%, the PSPP-CNN is considered to be achieving convergence. 

## 3. Experiment

To verify the validity of the proposed method, two series of experiments are presented in this paper. Fault diagnosis of constant and variable rotating speed data are conducted using the proposed method.

### 3.1. Constant Rotating Speed Data

**Figure 7:**
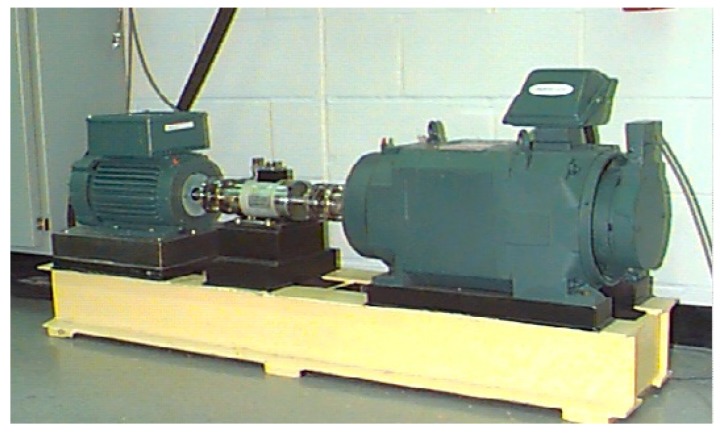
Bearing test stand used by Case Western Reserve University (CWRU).

The bearing fault data from the Case Western Reserve University (CWRU) Bearing Data Center [37] is selected to verify the validity of the method in a constant rotating speed environment. The bearing test stand used in the experiment is shown in Figure 7. There are four bearing states: normal, ball fault, inner race fault, and outer race fault. In each bearing fault state, the bearings have fault diameters of 0.007 inches, 0.014 inches, and 0.021 inches. There are also 0.028 inches fault data of ball fault and inner race fault. Thus, there are 12 conditions in total. The fault bearings are installed on the drive end. Three accelerometers are installed on the fan end, drive end, and the base, respectively. The rotating speed of the shaft is about 1800 rpm with the motor load ranging from 0 to 3 HP. All the data selected were sampled at a frequency of 12 kHz.

In our fault diagnosis experiment, all the data are divided into 12 conditions according to the fault states and fault diameters. The influences of fault bearing location, accelerometer location, and motor load are ignored. Because the data were stored as a long array with more than 250,000 data points, the data were divided into several samples. Each sample contains 1024 data points. The size of each condition used for training set and test set are listed in Table 3. The selection of training samples is random. The ratio of training samples to test samples is 2 to 1.

Because the sampling frequency of all data is the same, and the change in rotating speed is very small, it can be considered that the sampling frequency of all data is equal to the same multiple of the rotating frequency, namely a 400 multiple. At the same time, because the characteristic frequency of these bearing faults is higher than a two multiple of the rotating frequency, in this experiment, the continuous wavelet transform of the bearing data is carried out from 1 to 200 scales. Hence, 200 × 200 CWTSs are obtained as the CNN input.

To compare the diagnosis effectiveness of the three networks, CNN, SPP-CNN, and PSPP-CNN, on constant rotating speed data, three models are built to diagnose the samples. The structures of the CNNs are listed in Table 4:

As shown in Table 4, the first five layers of the CNNs are set as the same. Because the SPP layer and PSPP layer can reduce more image size than the max pooling layer, the original CNN has more layers and more connections. PSPP-CNN has two more convolutional layers than SPP-CNN for further feature exaction. The selection of CNNs parameters is problem dependent and obtained by trial and error. The selection of the parameters of the first 5 layers was based on the principles proposed in [38]. Then a validation set was built to optimize the parameters of the layers after layer 5 in three networks. The parameters that have best performance on validation set were chosen as the final CNN parameters. The training rate of all three CNNs is set to 0.002 and changed to 0.0005 when the training error is reduced to 1%.

MATLAB is used to implement the training on the computer with two E5-2667 v3 CPUs, a GTX1080Ti GPU, 32 GB memory, and a 1 TB driver. Based on Matconvnet toolkit [39], the SPP-CNN and PSPP-CNN layer are implemented by adding new layer types to it. After the convergence of the CNNs, the test samples are sent into CNNs for fault diagnosis. The convergence and accuracy of CNNs are listed in Table 5:

From Table 5, it is clear that the accuracy of SPP-CNN is a bit lower than that of CNN in Constant rotating speed data classification. SPP-CNN may lose some important information during SPP layer which is a big feature reduction. However, the convolutional layer following a PSPP layer can re-extract fault features. PSPP-CNN has a diagnosis accuracy similar with the CNN method, and better than SPP-CNN. It has a much shorter training time of each steps, for it has much less parameters to be trained. This shows that the PSPP layer retains the main fault features while reducing the total number of features. The fault diagnosis accuracy of PSPP-CNN in 12 conditions is shown in Table 6. All the conditions have an accuracy greater than 91.03%. Compared with diagnosis accuracies listed in [37], PSPP-CNN has equivalent accuracy, lower proportion of training samples and more conditions. This shows that the method we proposed has good performance in a constant rotating speed data diagnosis. 

### 3.2. Variable Rotating Speed Data

The fault diagnosis of equipment with variable rotating speed is an important issue that has not been solved satisfactorily. The proposed method is capable of handling variable rotating speed data. Therefore, a variable rotating speed experiment is carried out to show its advantages. 

**Figure 8:**
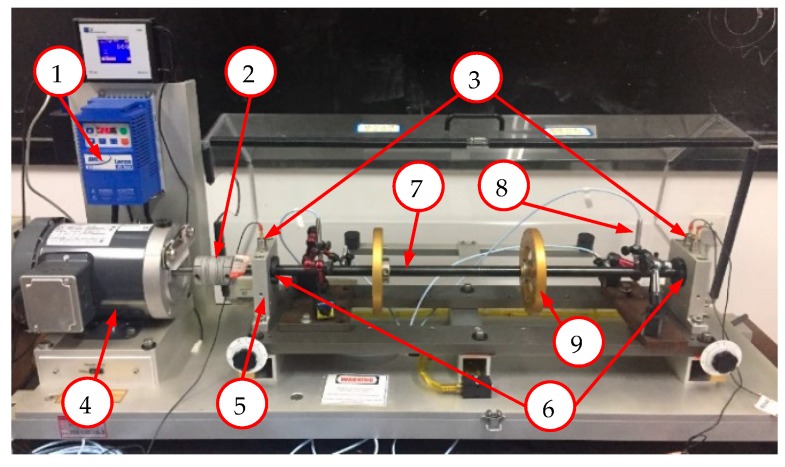
Machinery Fault Simulator-Rotor Dynamics Simulator (MFS-RDS) test bed. It can be used to simulate shaft, motor and bearing faults. The eddy current sensors are used to monitor the state of shaft. The data of them are not used for bearing diagnosis experiment. (1) speed controller, (2) rigid coupling, (3) accelerometer, (4) electromotor, (5) bearing base, (6) bearing, (7) shaft, (8) eddy current sensor, (9) rotary table.

The test bed used in this experiment, the Machinery Fault Simulator-Rotor Dynamics Simulator (MFS-RDS), is shown in Figure 8. Bearing fault experiments were conducted on this test bed. The bearing used is ER-16K LINK-BELT (LBX Company LLC, Lexington, KY, USA). There are four bearing fault modes: normal (NO), ball fault (BA), inner race fault (IR), and outer race fault (OR). The fault bearings can be installed at the drive or non-drive end, and each experiment has at most one fault bearing. Two accelerators are installed on the vertical direction of the two bearing housings. The load is constant in the experiment. Under each bearing fault condition, three sets of data are collected at the rotating speed of 1800 rpm, 2400 rpm, and 2900 rpm, respectively, and the sampling time of each set is approximately 10 min. The sampling frequency of all data is 12 kHz.

In the fault diagnosis experiment, the data are divided into 12 cases according to the fault status and rotating speeds. The influence of fault bearing position and sensor position is neglected. Because the data are stored as continuous time series, the data are divided into several samples. Each sample contains 1200 data points. Next, 5376 samples are obtained in each case; half of them, 2688 samples, are used for training, and the remaining 2688 samples are for test. The ratio of training samples to test samples is 1:1. Therefore, we have 32,256 training samples and 32,256 test samples totally. The fault diagnosis aims to classify the data into four categories based on the fault status.

Because all data samples have the same sampling frequency of 12 kHz, this corresponds to 400, 300, and 248 multiples of the rotating speed at 1800 rpm, 2400 rpm, and 2900 rpm, respectively. Thus, the continuous wavelet transform of three sets of bearing data is carried out from 1 to 400 scales, 300 scales, and 248 scales, respectively. In the time axis, the middle 400, 300, and 248 coordinates are chosen, because they have data points of one rotating period and can neglect the first and last few points of each sample. Hence, the CWTSs are chopped to square CWTSs that have a size of 400 × 400, 300 × 300, and 248 × 248, respectively.

The PSPP-CNN structure used in this experiment is shown in Figure 4 and Table 4. There are five convolutional layers, three max pooling layers, one PSPP layer, and one fully connected layer. The training rate is initially set to 0.002 and changed to 0.0005 when the training error is reduced to 1%. The training environment is the same as the constant rotating speed data training. It takes 317 min to achieve convergence after 44 training steps which means the error of training samples is less than 0.1%.

The confusion matrix of fault diagnosis result is shown in Table 7. The first row represents the rotating speed and labels of the test data. The first shows the diagnosis result labels. The method has a high diagnosis accuracy of 99.11%, and an accuracy of more than 97.61% is obtained for the data of each fault condition. This indicates that the PSPP-CNN method is suitable for bearing fault diagnosis with variable rotating speed.

**Figure 9:**
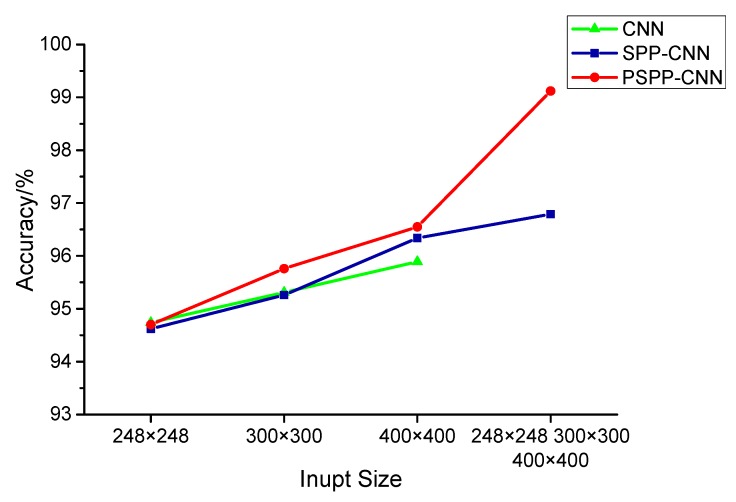
Accuracy of test samples using different input size and CNN models.

To compare the diagnosis effect with CNN and SPP-CNN, two other models are built using the CNN and SPP-CNN structures, as listed in Table 4. As CNN can only accept fixed-size images, all the data to 400 × 400, 300 × 300, and 248 × 248 CWTSs are translated to train the CNN separately. Accordingly, the CNN structure changes by adding a convolutional layer of a different size before the fully connected layer. SPP-CNN uses the same input images as those used by PSPP-CNN. 400 × 400, 300 × 300, and 248 × 248 CWTSs are also used to train SPP-CNN and PSPP-CNN. The diagnosis accuracy of seven different CNN models are listed in Table 8 and Figure 9.

As shown in Table 8, PSPP-CNN has a better diagnosis accuracy than other CNN and SPP-CNN models with single-size or multi-size input. Figure 9 shows that the accuracy increases along with the size and diversity of input samples. The method we propose using PSPP-CNN and multi-size input has the best diagnosis accuracy 96.58%. It shows a nearly two-percent improvement over SPP-CNN. The SPP-CNN model with multi-size does not get a big accuracy improvement. It is because that different from image recognition tasks, fault diagnosis needs precisely feature positioning in CWTS. A SPP layer before fully connect layer will lose more position information in CWTS than a PSPP layer which can locate as front layer of PSPP-CNN. In addition, using the normal CNN, the diagnosis accuracy increases with the increase of input image size. According to our analysis, this is mainly owing to the increase of 1800 rpm data accuracy which are 92.05%, 94.22%, 95.53% separately. The reason may be that the 300 × 300 and 248 × 248 cropping of CWTSs will lose some of the fault features of 1800 rpm data. An input size larger than 400 × 400 will not increase the accuracy.

To compare this PSPP-CNN method with other CNN-based fault diagnosis methods, several proposed methods are applied using the same dataset. Deep Convolution Neural Network with Wide first-layer kernels (WDCNN) [12] is a bearing diagnosis method that uses raw vibration signals as input, and wide kernels in first layer for feature extraction and high-frequency noise suppression. Dislocated Time Series Convolutional Neural Network (DTS-CNN) [18] is proposed for mechanical signals by adding a dislocate layer to CNN. DTS-CNN can extract the relationship between signals with different intervals in periodic mechanical signals. Resample-CNN [38] uses a resample method to normalize the data to make sampling frequencies are the same multiples of the rotating frequency. The three proposed methods are used to compare the fault diagnosis accuracy using the same dataset, for they are all proposed for the diagnosis of machinery using vibration signals based on CNN. Because the data used in the papers have similar testbed structure, sampling frequency and rotating speed, WDCNN and DTS-CNN use the same network parameters as those in the papers. Resample-CNN uses the 400 × 400 CNN structure for they have the same input size. As all the three methods use the ordinary CNN and have the similar CNN structures, the changes of CNN parameters will not change the diagnosis result greatly. What we need to focus on is the construction of deep learning input. As the accuracies shown in Table 9, the method proposed in this paper has the best performance in the diagnosis of variable rotating speed data.

The reason for the comparison made on this set of data is that the accuracies on this case can reflect the effectives of the methods both on constant speed and variable speed data. In addition, in practical use, most variable speed machines work like this case at a certain speed range or some preset optimal speed points. 

**Figure 10:**
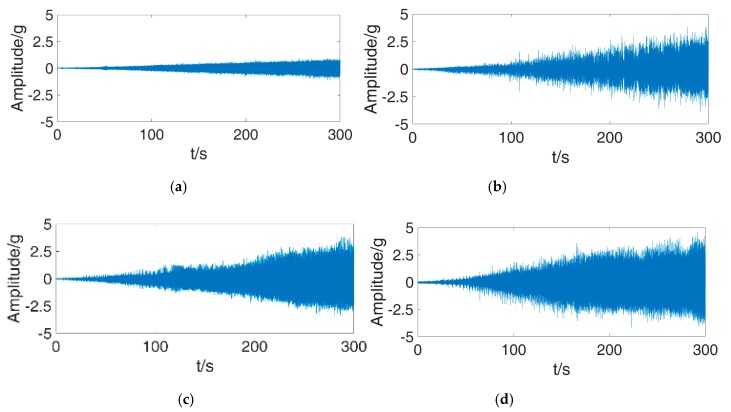
Vibration signals of four fault conditions. (**a**) normal (**b**) ball (**c**) inner race (**d**) outer race.

To further study the effectiveness of the method at full working speed range, an experiment to diagnosis the full working speed data is carried on using the PSPP-CNN trained above. The data of four bearing conditions were collected with 12 kHz on MFS-RDS. The fault bearing was installed on drive end. The rotation speed ranges from 300 to 3000 r/min. The rotation frequency increases at 0.15 Hz/s. Figure 10 shows the vibration signals of drive end accelerator in four fault conditions. 

To test the PSPP-CNN trained, the data of drive end accelerator in each condition are divided into 440 samples. Each sample contains 8192 data points. We get the cropped CWTS of each sample according to rotating speed. Therefore, the size of cropped CWTSs are ranges from 240 × 240 to 2400 × 2400. Then the cropped CWTSs are sent into PSPP-CNN for fault diagnosis. The accuracy of each fault condition is listed in Table 10.

As shown in Table 10, PSPP-CNN has diagnosis accuracies more than 90% for each fault conditions. It means that the PSPP-CNN trained by data at some rotating speed can be used to diagnosis bearing fault in full working speed. Through analysis, the accuracy of data under 1200 rpm is a little lower. Adding an incremental training using low speed data will increase the accuracy. It shows that the PSPP-CNN trained using data of few certain rotating speeds can be used to diagnose bearing fault in full working speed.

Through the fault diagnosis experiments of constant and variable rotating speed data, we can know that the PSPP-CNN method proposed is an effective solution for fault diagnosis of bearing. When applied to intelligent diagnosis system, the method has some advantages. First, the PSPP-CNN proposed in this paper can be easily implemented by adding a PSPP layer to the ordinary CNN code based on max pooling layer. There are some mature CNN frameworks based on MATLAB, Net Framework or Python. All of them can be easily built. Second, the fault diagnosis process has high power efficiency. As shown in Table 5, PSPP-CNN has less parameters than an ordinary CNN with the same front layers. It reduces the computation of each training and test epoch. Although the training of PSPP-CNN is still time-consuming in the computer without GPU accelerated computing, the diagnosis process using trained PSPP-CNN model can be completed quickly even at laptop computer. Third, with the de-noising ability of wavelet transform, the diagnosis system has good robustness. Diagnosis result will not be affected by the background noise in signal of practical equipment.

In practical applications, we can gather the experiment data or online monitoring data from the varying working speed bearing and use the data to train PSPP-CNN for fault diagnosis. The intelligent fault diagnosis method proposed in this paper has been used for online fault diagnosis of wind turbine bearings in a wind farm. The fault diagnosis software is exploited by C#& MATLAB combined programming. The signal is transmitted to MATLAB for CWTS calculation and classification. The diagnosis result can be obtained in 5 s. The training data was collected from the online vibration monitoring system installed on wind turbines. The fault data was picked out by referencing fault records.

## 4. Conclusions

In this paper, we propose an intelligent fault diagnosis method for a variable rotating speed bearing. The proposed approach is built upon CWTS and PSPP-CNN. This method decomposes vibration signals of bearing into CWTSs of different scales according to the rotating speed. In addition, the different size CWTSs are sent into PSPP-CNN for fault diagnosis. The PSPP-CNN that we proposed is an improvement of the SPP-CNN. The PSPP layer can fully use the pooling result for further feature extraction than SPP layer as they all can pool the input of different sizes to a fixed size. A series of experiments are carried out using constant rotating speed data and variable rotating speed data. The results show that the proposed approach is an effective solution. The method has been used in practical applications.

Although many fault diagnosis methods based on deep learning have been proposed, most methods are totally data-driven and focus on the small improvement of deep learning algorithm. The domain knowledge that has been used for fault diagnosis in recent decades is barely used. In addition, the working condition information is not considered in the input of deep learning algorithm. This paper takes the fault characteristics frequency of bearing and working speed into consideration. However, more domain knowledge and working condition information, such as load and output power, can be combined with deep learning. It may improve the accuracy and robustness, and the features extracted will be more interpretable. 

## Figures and Tables

**Table 1:** Parameters of a 4-level SPP.

Input Size	Level	Filter	Stride	Output Size	Output Length
15 × 15	1	15	15	1 × 1	30
	2	8	7	2 × 2	
	3	5	5	3 × 3	
	4	4	3	4 × 4	
20 × 20	1	20	20	1 × 1	30
	2	10	10	2 × 2	
	3	7	6	3 × 3	
	4	5	5	4 × 4	

**Table 2:** Convergence time and accuracy using two back-propagation methods.

Method	Training Steps	Convergence Time/Min	Time of Each Step/Min	Accuracy/%
1	63	324	5.14	92.43
2	51	263	5.16	92.52

**Table 3:** Sizes of training and test sets in 12 conditions.

Fault	None (NO)	Ball (BA)				Inner Race (IR)				Outer Race (OR)			Total
Diameters/in	0	0.007	0.014	0.021	0.028	0.007	0.014	0.021	0.028	0.007	0.014	0.021	
Training set size	24720	10080	10080	10080	3360	10080	10080	10080	3360	30240	10080	30240	162480
Test set size	12360	5040	5040	5040	1680	5040	5040	5040	1680	15120	5040	15120	81240

**Table 4:** Structure of three Convolutional neural networks.

Layer	1	2	3	4	5	6	7	8	9	10	11
CNN	Conv 5 × 5 × 1 50	MaxPool 2 × 2	Conv 5 × 5 × 50 50	MaxPool 2 × 2	Conv 4 × 4 × 50 100	MaxPool 2 × 2	Conv 5 × 5 × 100 100	MaxPool 2 × 2	Conv 4 × 4 × 100 200	MaxPool 3 × 3	FC
SPP-CNN	Conv 5 × 5 × 1 50	MaxPool 2 × 2	Conv 5 × 5 × 50 50	MaxPool 2 × 2	Conv 4 × 4 × 50 100	MaxPool 2 × 2	Conv 5 × 5 × 100 100	SPP 5	FC		
PSPP-CNN	Conv 5 × 5 × 1 50	MaxPool 2 × 2	Conv 5 × 5 × 50 50	MaxPool 2 × 2	Conv 4 × 4 × 50 100	PSPP (8,6)	Conv 5 × 5 × 100 100	MaxPool 2 × 2	Conv 3 × 3 × 100 200	FC	

**Table 5:** Convergence and accuracy of Convolutional neural networks.

Model	Number of Parameters	Training Steps	Convergence Time/Min	Accuracy/%
CNN	1.1e6	38	208	97.86%
SPP-CNN	1.5e6	48	281	97.23%
PSPP-CNN	5.8e5	44	211	97.79%

**Table 6:** Accuracy of test samples in 12 conditions using Pythagorean Spatial Pyramid Pooling Convolutional Neural Network (PSPP-CNN).

Fault	None	Ball				Inner Race				Outer Race			Total
Diameters/in	0	0.007	0.014	0.021	0.028	0.007	0.014	0.021	0.028	0.007	0.014	0.021	
Accuracy/%	99.98	95.32	95.99	91.03	99.64	99.94	94.17	99.01	99.88	98.09	96.96	99.31	97.79

**Table 7:** Accuracy of test samples at different rotating speeds using PSPP-CNN.

Labels	NO 1800 rpm	IR 1800 rpm	OR 1800 rpm	BA 1800 rpm	NO 2400 rpm	IR 2400 rpm	OR 2400 rpm	BA 2400 rpm	NO 2900 rpm	IR 2900 rpm	OR 2900 rpm	BA 2900 rpm	Accuracy %
NO	2631	0	0	48	2686	1	0	17	2688	3	0	31	99.27
IR	0	2687	7	0	0	2685	5	3	0	2678	8	25	99.83
OR	0	1	2681	0	0	2	2683	0	0	7	2680	69	99.75
BA	57	0	0	2640	2	0	0	2668	0	0	0	2563	97.61
Accuracy%	97.88	99.96	99.74	98.21	99.93	99.89	99.81	99.26	100	99.63	99.70	95.35	99.11

**Table 8:** Accuracy of test samples using different CNN models.

Input Size	CNN	SPP-CNN	PSPP-CNN
248 × 248	94.74	94.62	94.70
300 × 300	95.31	95.26	95.76
400 × 400	95.89	96.34	96.55
400 × 400, 300 × 300, 248 × 248		96.79	99.11

**Table 9:** Fault diagnosis result using other proposed CNN-based methods.

Model	Deep Convolution Neural Network with Wide first-layer kernels	Dislocated Time Series Convolutional Neural Network	Resample-CNN	PSPP-CNN
Accuracy/%	97.76	96.20	98.15	99.11

**Table 10:** Accuracy of four fault conditions at full rotating speeds using PSPP-CNN.

Fault	None	Ball	Inner race	Outer race	Total
Accuracy/%	90.23	91.82	92.05	92.95	91.76
